# B16F0 melanoma exosomes deliver a unique and complex biological payload that includes Ptpn11 to suppress T lymphocyte function

**DOI:** 10.1186/2051-1426-3-S2-P270

**Published:** 2015-11-04

**Authors:** Yueting Wu, Wentao Deng, Emily McGinley, David J Klinke

**Affiliations:** 1 West Virginia University, Morgantown, WV, USA

## Background

Host immunity is coordinated through a complex network of intercellular communication. Similar to the sculpting of tumor antigens during oncogenesis, a related hypothesis is that proteins secreted by malignant cells are shaped by somatic evolution. Malignant cells that then emerge secrete proteins that alter intercellular communication to promote tumor growth. As exosomes represent an emerging mode of cell-to-cell communication through the delivery of proteins and coding and non-coding RNAs, the objective of this study was to test this hypothesis by characterizing the impact of exosomes derived from three melanocyte cell lines on T cell function, with an emphasis on exosomal mRNA.

## Methods

We analyzed exosomes from three melanoma models: B16F0, a non-immunogenic model of malignant melanoma; Cloudman S91, a model of immunogenic melanoma; and Melan-A, an immortalized melanocyte cell line.

## Results

Using electron microscopy, exosomes derived from all three cell lines were morphologically similar and uniformly distributed in size. With a median size of 160 nm in diameter, exosomes were sized to remain within the tumor. The exosomes contained receptors derived from the parent cell as demonstrated by IL12RB2 expression on B16F0 exosomes and intact mRNAs. Furthermore, transcript profiling of B16F0 exosomes and cells suggested that exosomal mRNA is enriched for mRNAs that target immune-related pathways, including Ptpn11 that inhibited T cell proliferation and Dnmt3a that inhibited T cell production of IFN-gamma. Functionally, B16F0 exosomes dose-dependently suppressed cell proliferation and the expression of IL12RB2 in primary CD8+ T cells. In contrast, Cloudman S91 exosomes promoted T cell proliferation and Melan-A exosomes had a negligible effect on primary CD8+ T cells.

## Conclusion

Collectively, the results are consistent with somatic editing of exosomal payloads and suggest that exosomes establish a density-dependent field effect by altering the activity of immune cells that enter the tumor microenvironment.

